# Validation of A Wireless Telemetric Bladder Pressure Monitoring System in Traumatic Thoracic Spinal Cord Injury in Yucatan Minipigs

**DOI:** 10.1177/08977151251389956

**Published:** 2025-11-14

**Authors:** Adam W. Doelman, Jay Ethridge, Femke Streijger, Audrey Warner, Megan Webster, Avril Billingsley, Sid Gunamalai, Kitty So, Husain Kankroliwala, Martin S.M. Keung, Neda Manouchehri, Alex Kavanagh, Steve J.A. Majerus, Margot S. Damaser, Brian K. Kwon

**Affiliations:** 1 International Collaboration on Repair Discoveries, University of British Columbia, Vancouver, Canada; 2 Department of Electrical, Computer, and Systems Engineering, Case Western Reserve University, Cleveland, Ohio, USA; 3 Advanced Platform Technology Center, Louis Stokes Cleveland VA Medical Center, Cleveland, Ohio, USA; 4 Department of Biomedical Engineering, Lerner Research Institute, Cleveland Clinic, Cleveland, Ohio, USA; 5 Department of Orthopaedics, Vancouver Spine Surgery Institute, University of British Columbia, Vancouver, BC, Canada

**Keywords:** neurogenic bladder, pig, porcine model, spinal cord injury, telemetry, urodynamics

## Abstract

Neurogenic lower urinary tract dysfunction (NLUTD) is a major cause of morbidity and reduced quality of life after spinal cord injury (SCI). In pre-clinical research, small and large animal models such as rats, dogs, and minipigs have been used to investigate NLUTD through urodynamic studies (UDS) such as conventional filling cystometry. Although filling cystometry is currently considered the gold standard for bladder monitoring in pre-clinical research, this approach has several well-recognized limitations. The aim of this study was to develop and evaluate the feasibility of an implantable, radiotelemetric system for monitoring bladder pressure in a Yucatan minipig model of SCI. The transmitter was surgically implanted in the dome of the bladder and several UDS experiments were conducted to evaluate the system’s effectiveness at measuring pressure compared to conventional UDS equipment. We observed a strong correlation and agreement between the transmural telemetry sensor and the UDS system. There was no significant difference between bladder compliance and baseline bladder pressure between the two sensor systems. However, the telemetry system recorded significantly lower voiding and non-voiding contraction pressure amplitudes as well as lower voiding threshold pressures and detrusor after-contraction measured with the telemetry system. The telemetry system appeared to be a reliable and accurate method for assessing bladder pressure and allowed for an evaluation of urodynamics in a pig model of SCI for several months. The application of this method could enable a more detailed in vivo evaluation of NLUTD after SCI and a better understanding of micturition behavior during natural-filling, ambulatory urodynamics.

## Introduction

Neurogenic lower urinary tract dysfunction (NLUTD) is a common secondary complication of traumatic spinal cord injury (SCI) and a significant cause of morbidity for those living with SCI.^1^ NLUTD, as a result of traumatic SCI, can include a multitude of secondary complications, placing a considerable financial, psychological, and social burden on individuals living with this condition.^2,3^ For instance, impairment of voiding and bladder emptying, particularly the dyssynergic activation of detrusor and external urethral sphincter (EUS) muscles, can result in the accumulation of high pressure within the urinary tract, which, if not properly managed, can irreversibly damage renal tissue.^4^

Many studies have been published on NLUTD after SCI in rodent models, given the well-established techniques for inducing SCI and sustaining animals post-injury with neurological impairments.^5-8^ The minipig has been described more recently as a model of SCI,^9-13^ and its size and anatomical and physiological similarities to the human lower urinary tract (LUT)^14-16^ make the minipig an attractive intermediary large animal model for studying NLUTD. Furthermore, in our recent work, we found that the pathophysiological changes in bladder pressure patterns in awake minipigs after SCI closely resemble those seen in human post-SCI urodynamic studies (UDS).^17^

Although performing awake cystometry in the minipig model of SCI has provided us with a unique opportunity to serially evaluate NLUTD over time after SCI, cystometry has inherent limitations. Fundamentally, conventional cystometry depends upon an artificial retrograde faster-than-physiological filling of the bladder, which may not produce results representative of the physiological state of the LUT.^18,19^ Furthermore, cystometry only captures a single snapshot of LUT function (or dysfunction) at that given point in time, which may not fully represent what is occurring in the normal ambulatory setting.^20,21^

Ambulatory urodynamic monitoring can overcome the shortcomings of retrograde filling cystometry.^18^ This involves monitoring the bladder pressure over a period of time while the patient is in their usual environment. Previous studies have demonstrated that radiotelemetric monitoring of LUT function is possible in neurologically intact dogs and minipigs.^22^ However, the presence of neurological impairment caused by severe traumatic SCI adds additional complexity and challenges to consider, such as potentially high external forces exerted upon the bladder by dragging of the rump, which can cause physical harm to the monitoring system.

Therefore, the primary aim of this study was to establish surgical techniques for implanting a telemetry system for bladder pressure monitoring in the Yucatan minipig model of SCI^17,23^ and then to evaluate its accuracy at measuring bladder pressure before and after thoracic SCI in comparison to conventional catheter-based cystometry.

## Materials and Methods

The data presented in this study were collected at the University of British Columbia (UBC), Vancouver, British Columbia, Canada. All animal protocols and procedures were approved by the Animal Care Committee of UBC and were in accordance with the Canadian Council on Animal Care (CCAC).

### Telemetric pressure monitoring system

**FIG. 1.**
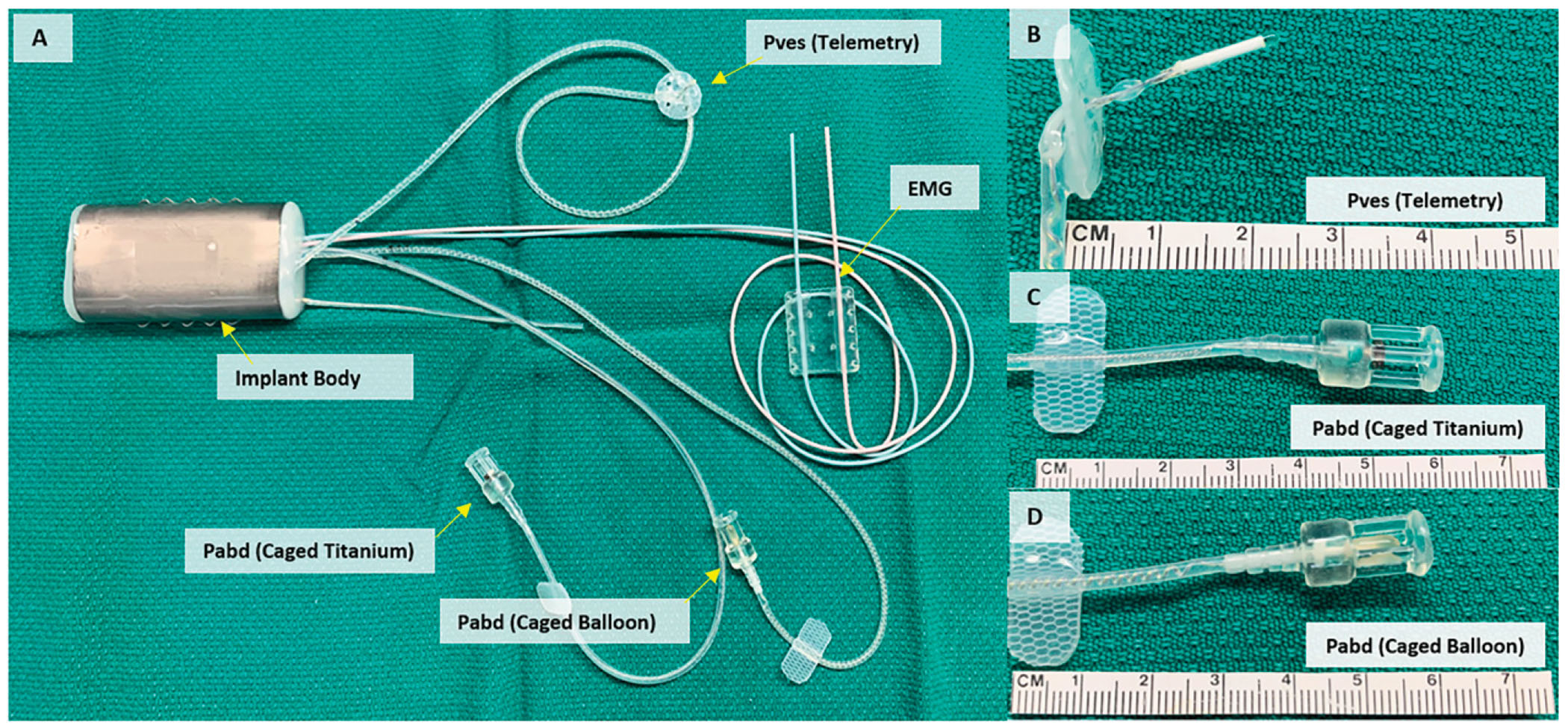
Telemetric implant overview. **(A)** Device overview showing the complete implant, including the body (transmitter), transmural Pves sensor, Pabd Caged Titanium and Caged Balloon sensors, and EMG leads, along with the silicone endplate, which lies under the EUS after implantation. **(B)** Pves transmural pressure probe. Design includes a suture ring and a 2 cm pressure-sensing glass probe; **(C)** Pabd Caged Titanium consisting of a titanium pressure sensor encased in a hard silicone exterior cage for added durability; **(D)** Pabd Caged Balloon design consists of a glass pressure sensor contained within a silicone balloon all encased in a hard silicone cage. EMG, electromyographic; EUS, external urethral sphincter; Pves, bladder pressure; Pabd, abdominal pressure.

The Stellar telemetry system consists of an implantable transmitter unit integrated with three solid-state pressure-tipped sensors (1× vesicular, 2× abdominal) and electromyographic (EMG) fine wires (#PPPBTA-XXL; TSE Systems, Chesterfield, MO, USA) (Fig. 1). Internal components of the transmitter unit included a battery, a microprocessor, and an internal memory storage (1 GB). The transmural bladder pressure sensor (Pves Telemetry) consisted of a 2 cm glass-tipped sensor attached to a flexible silicone ring to permit attachment to the external bladder wall. The bladder sensor was also outfitted with a small silicone “stopper” ~1.5 cm from the sensor tip to improve its stability along the inner bladder wall.

The abdominal pressure (Pabd) sensor design initially consisted of a glass-tipped sensor embedded in a flexible silicone balloon, which was then encased in a rigid encapsulation cage. Significant durability challenges were encountered when used in the abdominal cavity. Ultimately, we overcame this issue by switching to a titanium-housed sensor while retaining the rigid silicone encapsulation cage (Pabd Caged Titanium). Each pressure transmitter arrived calibrated by TSE Systems in a closed system at several different temperature points to establish a pressure/temperature relationship with a final calibration to 37.5°C.

### Experimental design

**FIG. 2.**
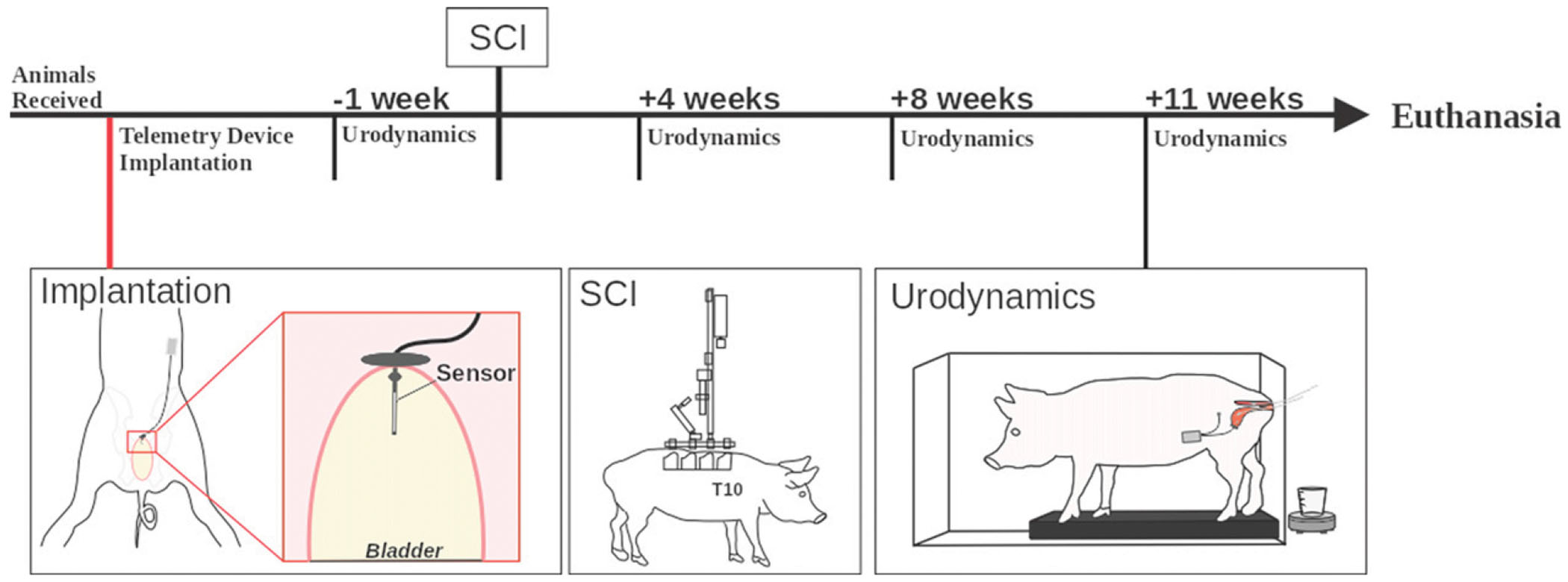
Experimental design depicting the implantation of the telemetric transmural bladder pressure sensor, spinal cord injury (SCI) surgery, and the urodynamics experiments conducted in Yucatan minipigs. Implantation was performed 5–7 weeks prior to SCI. Urodynamics were performed 1 week prior to SCI and 4, 8, and 11 weeks after experimental SCI.

Female Yucatan minipigs (*n* = 10) were obtained from Premier BioSource (formerly S&S Farms, California, USA), weighing ~12–15 kg upon arrival. After 1 week of acclimating to the facility, the animals began a 4- to 5-week sling training regimen to prepare for UDS as previously described.^17^ Surgical implantation of the telemetry implant was performed 5–7 weeks prior to SCI. Then, in each animal, filling cystometry was conducted 2 weeks before experimental SCI and repeated 4-, 8-, and 11-weeks post-SCI. Experimental end-point with tissue harvesting was performed 12 weeks post-SCI (17–18 weeks post-implantation). The pressure readings from the UDS catheters were compared with radiotelemetry values during urodynamic assessment. Urinalyses were performed at each cystometry experiment and at select times between experiments at the discretion of the attending veterinarian. We defined a clinically relevant UTI as an animal presenting with bacteriuria and pyuria on a standard urine culture test, as well as clinical symptoms including lethargy, malodorous urine, fever, and so forth. The overall experimental design is illustrated in Figure 2.

### Transmitter implantation surgery

**FIG. 3.**
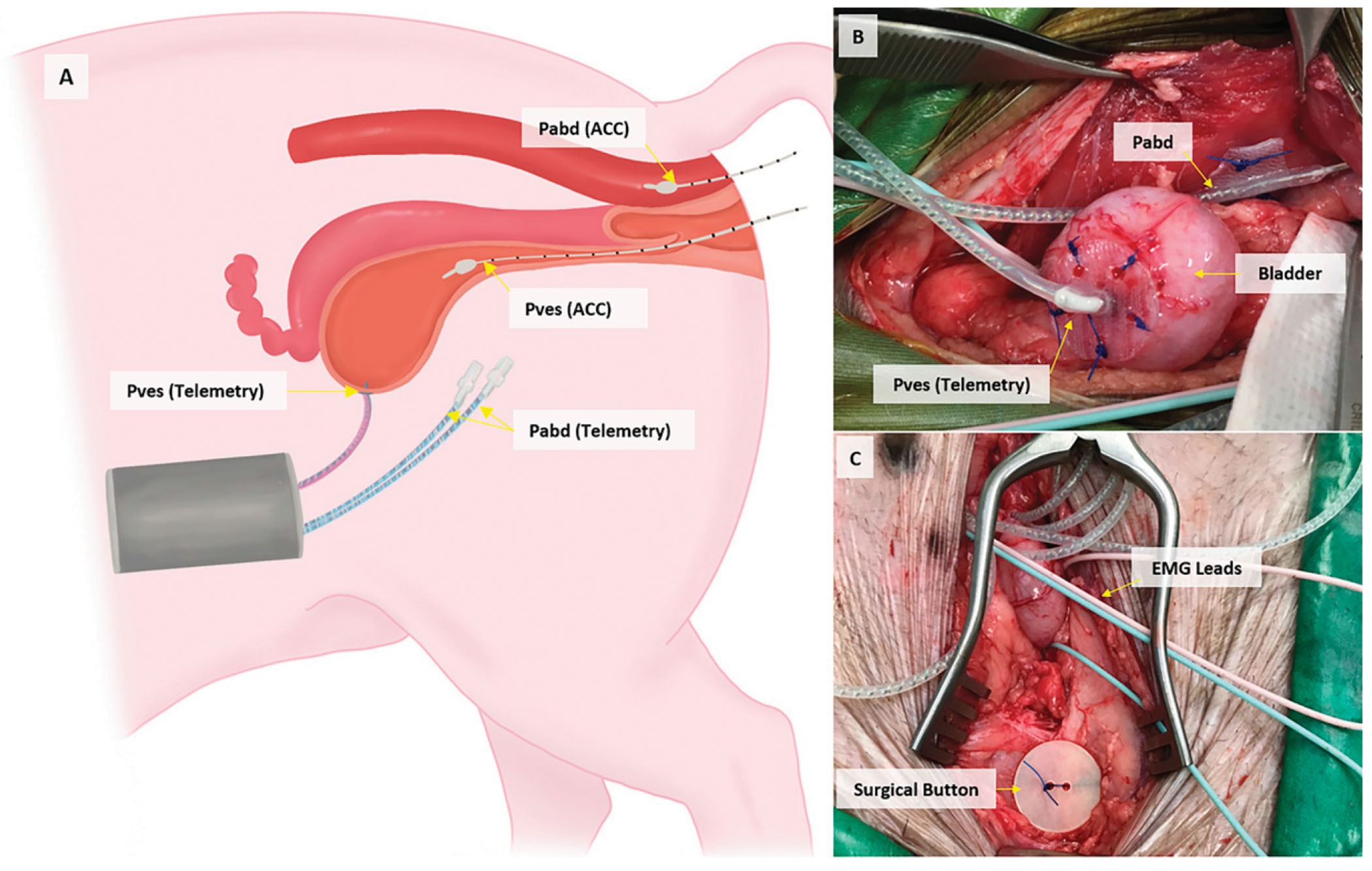
Implantation of the implanted radiotelemetric device in a Yucatan minipig. **(A)** Device consists of an implant body (transmitter), two abdominal pressure sensors (Pabd), one transmural pressure sensor (Pves), and electromyographic (EMG) leads to record external urethral sphincter (EUS) EMG activity. The device was validated against transurethral standard clinical air-charged catheters (ACC), Pves and Pabd. **(B)** Surgical implantation of the Pves Telemetry probe into the apex of the bladder with five sutures to attach the suture ring to the bladder wall. Pabd pressure sensor is also visible. **(C)** Implantation of EMG leads around the EUS and kept in place using a surgical button.

Telemetry implants were aseptically placed in all animals (Fig. 3). Animals were pre-anesthetized with an intramuscular (IM) injection of dexmedetomidine (0.02 mg/kg), midazolam (0.1 mg/kg), butorphanol (0.2 mg/kg), and atropine (0.02 mg/kg); endotracheally intubated; and maintained with isoflurane (2–2.5%) and fentanyl (3–7 mcg/kg/h) for the entire procedure, as previously described.^12,23^ An additional round of sterile cleaning was performed to ensure sterility around the incision sites. Animals were placed supine, and a midline incision was made to expose the peritoneal cavity. The pressure transducer was inserted in the dome of the urinary bladder, and a silicone ring attached to the bladder pressure probe was secured to the bladder wall with 4-0 taper Prolene sutures (Fig. 3B). After inserting the Pves probe, 60 mL of warm saline was infused into the bladder lumen to ensure proper adherence to the bladder wall with no leakage and to assess pressure transducer function. Two Pabd sensors were sutured to the parietal peritoneal surface using a 1 cm × 2 cm silicone “pad” along the connecting wire between the implant body and sensor, located 4 cm proximally relative to the sensor tip (Fig. 3C, D). The peritoneal, muscle, and fascia layers were then closed separately (2-0 taper Prolene suture) with the probes exiting caudally from the muscle layer. The subcutaneous fat and fascia layer were closed using a simple continuous suture pattern (2-0 taper PDS II suture); this was done in two layers to reduce the tension on the final skin sutures.

A left flank incision was then made, and a subcutaneous pocket was created for the transmitter implant. The transmitter was tunneled from the abdominal incision to the flank pocket and then secured to the underlying muscle, and the incision was closed in two layers: continuous for the fascia (2-0 taper PDS II suture) and single-interrupted and cruciate sutures for the skin (2-0 taper Prolene suture). After observing previous transmitters migrate ventrally from their original position after surgery, a sterile fibrous mesh wrap (EG-ACC-POCKET-XL; Transonic, Ithaca, NY, USA) was placed around the implant body before securing it to the subcutaneous pocket, improving its adherence to surrounding tissue. The abdominal skin layer was closed using intradermal continuous sutures (2-0 reverse cutting PDS II suture).

After implantation of the telemetry system, animals were individually housed for 7 days. During this period, the animals received antibiotics (Baytril 10 mg/kg IM, once daily, 5 days) and an anti-inflammatory (Meloxicam 0.2 mg/kg IM, once daily, 3–6 days). Animals were also given a Fentanyl patch (12.5 mcg/h; ~3 days) (Pigs A and B) or Buprenorphine (0.005–0.0025 mg/kg/h IV; 1–2 days) (Pigs C–I). Fluid output and vitals were continuously monitored for each animal by experienced animal care technicians. Throughout the postoperative recovery period, weekly in-person recordings were performed to ensure the transmitter was responsive and the sensors were functioning.

### UDS—filling cystometry

We previously established a training and setup protocol to perform conventional UDS in awake, conscious female Yucatan minipigs with clinical urodynamics equipment.^17^ The software used to record and analyze the UDS data was UDS-120 and UDS-120 Goby (Laborie Medical Technologies Corp., Montreal, Québec, Canada). Telemetric recording was performed concurrently during the test.

Animals were sling trained for 4–6 weeks to familiarize them with the experimental setup and the testing room. On the day of the study, animals were first sedated for transurethral catheter insertion. Sedation was induced using dexmedetomidine (IM, 0.005 mg/kg), butorphanol (IM, 0.5 mg/kg), and midazolam (IM, 0.5 mg/kg) and maintained with O_2_ and isoflurane (0.5–4%), as needed. Animals were then brought into the radiology suite and placed in sternal recumbency. The vulva and the surrounding areas were cleaned using 5% Hibitane (chlorhexidine gluconate 4%; Partnar Animal Health Inc., Ilderton, ON, Canada) before inserting the urodynamic intravesical (Pves) and rectal (Pabd) pressure catheters (T-DOC^®^ air-charged catheters; Laborie Medical Technologies Corp.). Following catheter insertion, animals were transported to the urodynamics suite and placed in a hammock-style sling (The Panepinto Sling^™^; Panepinto & Associates, Masonville, CO, USA) with a custom-made canvas to raise and support the lower abdomen and hindlimbs of pre- and post-SCI animals. A harness designed for dogs (Coastal Pet Products Inc., Alliance, OH, USA) was attached to the pig and tied to the restraint cage for additional support and to help with animal placement. Restraints were tied around each limb and attached to the restraining cage with sufficient slack to permit moderate lateral stepping and squatting in pre-SCI animals. EMG patch electrodes (Cleartrode^™^; ConMed Corporation, Utica, NY, USA) were placed peri-anally at the 9 and 3 o’clock positions, and the ground EMG patch was placed on the left or right calcaneus.

Sedation was then reversed with atipamezole (0.2 mg/kg; IM), and animals were given 10 min to regain consciousness and demonstrate alertness. Then, ambient-temperature saline (0.9% NaCl) was infused into the bladder at a rate proportional to the animal’s weight (1 mL/min/kg). A flow meter (Laborie Medical Technologies Corp.) was placed below the animal, and urine was funneled into a collection cup by an attendant. In uninjured pigs, one fill-and-void cycle was recorded. In SCI animals, which did not void volitionally, we continued the UDS recording until four leaks were observed or until the infusion volume reached 1 L. The bladder fill was paused at each void and, in SCI animals, resumed until more contractions were observed. At the end of the UDS test, the equipment was detached, and the pigs were transported back to their respective pens.

### Experimental SCI

Thoracic SCI was induced 5–7 weeks after implantation of the telemetry system. A severe T10 contusion/compression injury was induced as previously described.^12,23^ Briefly, animals were anesthetized and placed in sternal recumbency. A laminectomy was then performed from T9 to T11 to expose the spinal cord. Injury was then inflicted by dropping a 50 g impactor from a height of 16.73 cm. Following the contusion impact, a 100 g weight was placed on top of the impactor for a total of 5 min to simulate a clinical contusion/compression injury. The weight drop apparatus was then removed, and the wound was closed in anatomical layers.

Following the procedure, animals were monitored closely for 4–6 days in their respective pens. Fentanyl constant rate infusion was administered immediately after surgery as needed at the discretion of the attending veterinary staff (IV, 0.1–2.5 mcg/kg/h) and later switched to a transdermal patch 12.5–25 mcg/h for 72 h. Prophylactic antibiotics were administered immediately following the procedure (IV, Baytril, 5 mg/kg). Meloxicam (PO or SQ, 0.2 mg/kg) was provided as needed. Metoclopramide was administered for at least 12 h post-SCI for all animals (IV, 0.02 mg/kg/h). Following surgery, an indwelling catheter, which was inserted prior to the procedure, was left inside the animal and connected to a urine collection bag for 4–6 days. The catheter was then removed, and the bladder was allowed to drain spontaneously for the duration of the study.

### Data post-processing

Data presented herein were recorded from the start of the bladder fill to 20–30 sec after the final voiding phase in all animals. Telemetric pressure data were down-sampled from 500 Hz to 20 Hz to match the frequency of the urodynamics pressure data for statistical analysis. Signal dropouts were filtered by adjusting the upper or lower output domain using the NOTOCORD-hem 4.4 software (NOTOCORD-hem^™^; Instem, PA, USA). EMG was not validated for the telemetry system and was therefore omitted from the present study.

### Data analysis

Detrusor pressure (Pdet) was calculated as Pabd subtracted from simultaneous vesical (bladder) pressure (Pves). Outcome variables were calculated for pressure values measured using the UDS and telemetry systems and included maximum voiding contraction pressure, maximum non-voiding contraction pressure, baseline pressure, threshold pressure to void, bladder compliance, and peak detrusor after-contraction pressure. Voiding and non-voiding contraction amplitudes were calculated using the difference in Pves from a point just prior to the contraction to the peak Pves of the contraction waveform. Voiding contractions included all pressure waveforms greater than Δ7 cmH_2_O in amplitude with a visual observation of urine leakage. Non-voiding contractions were defined herein as pressure waveforms greater than Δ7 cmH_2_O in amplitude (DPves) without a reflected change in Pabd pressure (assessed using UDS Pabd catheter) to distinguish contractions from movement artifact. Baseline pressure (in cmH_2_O) was considered the Pdet value at the initiation of bladder fill. Threshold pressure to void was calculated by assessing the difference in Pdet from the start of bladder fill to the point when urine was first observed exiting the vulva during urodynamic assessment. Compliance was calculated as the change in bladder volume (volume infused, mL) divided by the difference in Pdet from the start of bladder filling to a point just prior to the first observed bladder contraction waveform.

Pearson correlation coefficients were calculated to compare simultaneous UDS and telemetry pressure data. Correlation values were categorized in the following manner: zero (<0.1), weak (0.1–0.39), moderate (0.4–0.69), strong (0.7–0.99), and perfect (1.0).^24^ Bland–Altman analyses were used to evaluate the agreement between simultaneous UDS and telemetry pressures. 95% limits of agreement (mean difference ± 1.96 standard deviation of the differences) are presented. Maximum voiding and non-voiding contraction amplitudes were compared using a paired *t* test.

A signal-to-noise ratio was calculated for UDS and telemetry pressure readings. Signal was considered the average peak Pves of all voiding contractions recorded during a single UDS experiment (in cmH2O). Noise was the standard deviation of Pves values collected within a 50 sec window (1000 data samples) for each experiment. We determined the specific window used for noise calculation by selecting the 50 sec sample with a mean absolute value closest to zero (effectively the most “stable” baseline measurement) after applying a median trend removal. Signal-to-noise comparison was made between pressure sensors using a mixed-effects model analysis.

Statistical analyses were conducted using GraphPad Prism 10.2 (GraphPad Software, Inc., CA, USA) and MATLAB. Data are presented as mean ± standard deviation. Differences were considered to be statistically significant at *p* < 0.05.

## Results

### Telemetry device implantation—surgical considerations and tolerability over 13–18 weeks

A total of 10 pigs were studied, each receiving a telemetry implant 5–7 weeks prior to SCI surgery. SCI impact parameters include impulse, velocity at contact, cord displacement, ratio of displacement relative to velocity at contact, and zeroed force (Table 1).

Following device implantation, some animals developed a mild seroma around the transmitter site/flank incision; however, it appeared normal after 2 weeks in 9 out of 10 animals. Post-mortem examination of one animal euthanized 3 weeks post-implantation revealed an infection from the implant pocket along the connecting wires of the telemetry system into the peritoneum. This animal was excluded from the study. To reduce the risk of similar issues, we added a second sterile cleaning with a 5% Hibitane solution (chlorhexidine gluconate 4%; Partnar Animal Health Inc.) at the incision site before making the first incision, and we tunneled the telemetric transmitter from the abdominal incision site to the flank pocket, rather than vice versa. After these changes were implemented, no further infections were observed around the incision site.

In one case, migration of the transmural pressure sensor out of the bladder lumen despite suture fixation was observed at the experimental end-point. All telemetric intravesical pressure data collected from this animal (D) were therefore excluded from the study. Of the remaining nine animals, three were euthanized early due to the development of multiresistant UTI 13–14 weeks post-implantation. Since no UTIs were detected post-implantation and pre-SCI in any of the animals, we attributed the UTI to the SCI rather than to the presence of the implanted sensor. Table 2 summarizes the data included/excluded for each analysis presented herein.

### Durability of the telemetric bladder pressure monitoring system

In general, the telemetry sensors showed a high degree of functional stability in six out of nine animals for 13–18 weeks (longest experimental end-point). In one animal (C), the transmural bladder sensor failed <24 h after implantation; however, after receiving a replacement sensor during the SCI procedure, bladder pressure data from this animal were monitored from surgery until the experimental end-point. Supraphysiological (>200 cmH_2_O) intravesical pressure drift was recorded from the transmural bladder sensor in one animal (E) 4 weeks after SCI (10 weeks post-implantation). Additionally, in another animal (G), a malfunction of the transmural bladder sensor was observed 8.5 weeks post-SCI (14 weeks post-implantation), which resulted in data loss at that timepoint. The Pabd transmitter, which was housed in titanium, showed excellent durability for all experiments and was deemed appropriate for long-term *in vivo* studies.

### Performance assessment of the telemetric bladder pressure monitoring system

**FIG. 4.**
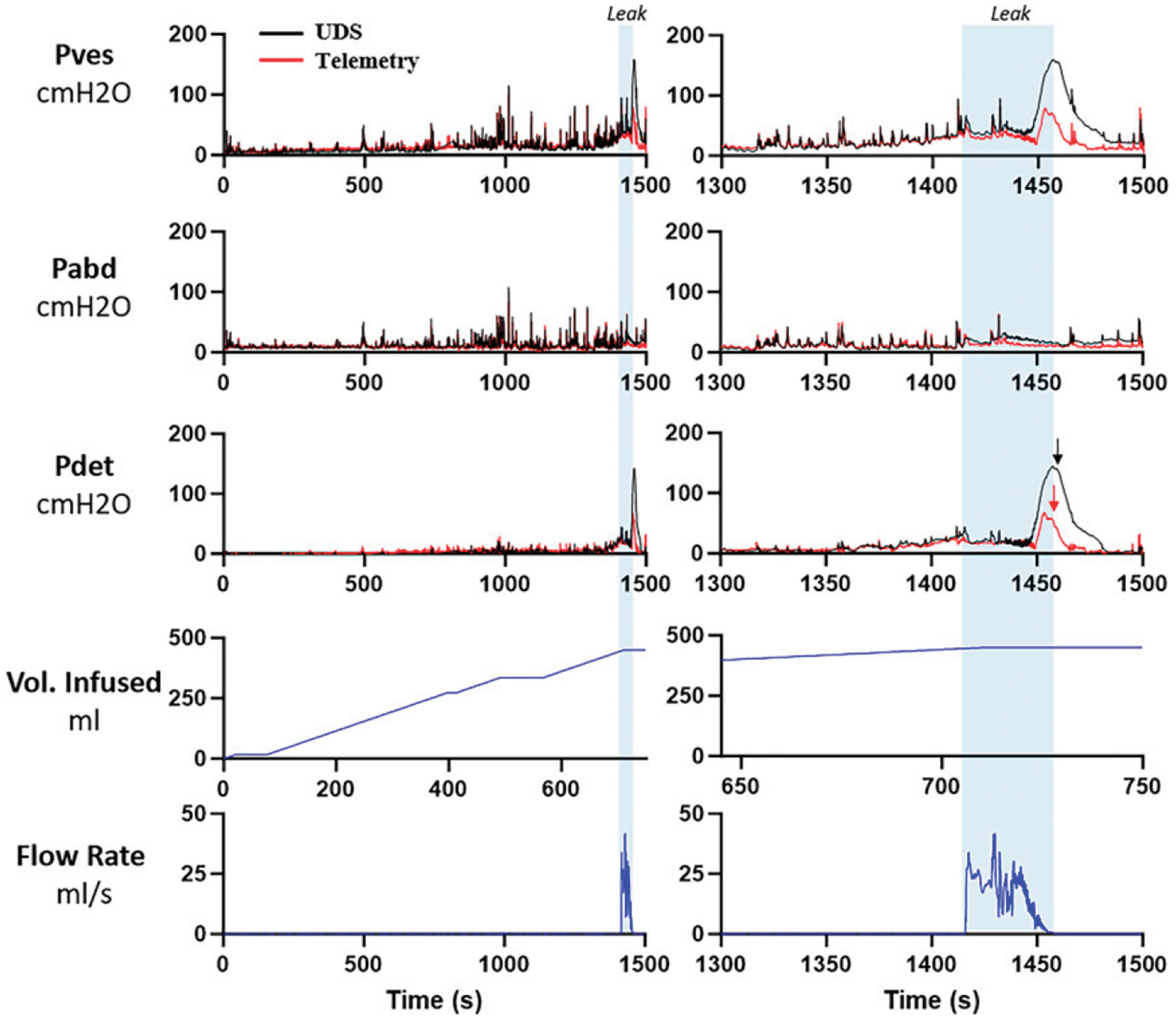
Example cystometrogram showing simultaneous air-charged catheter-based UDS pressures (black) and telemetric transmural and intra-abdominal pressure sensors (red) in a spinal-intact Yucatan minipig before spinal cord injury (SCI). One voiding contraction is presented herein and highlighted with blue shading. Right panels are a magnified range at the end of the full data set shown in the left panels. Black and red arrows are shown to highlight detrusor after-contraction in the Pdet UDS and Pdet Telemetry traces, respectively. Simultaneous volume infused (mL) and voided urine flow rate (mL/sec) are also included (dark blue). UDS, urodynamic studies.

**FIG. 5.**
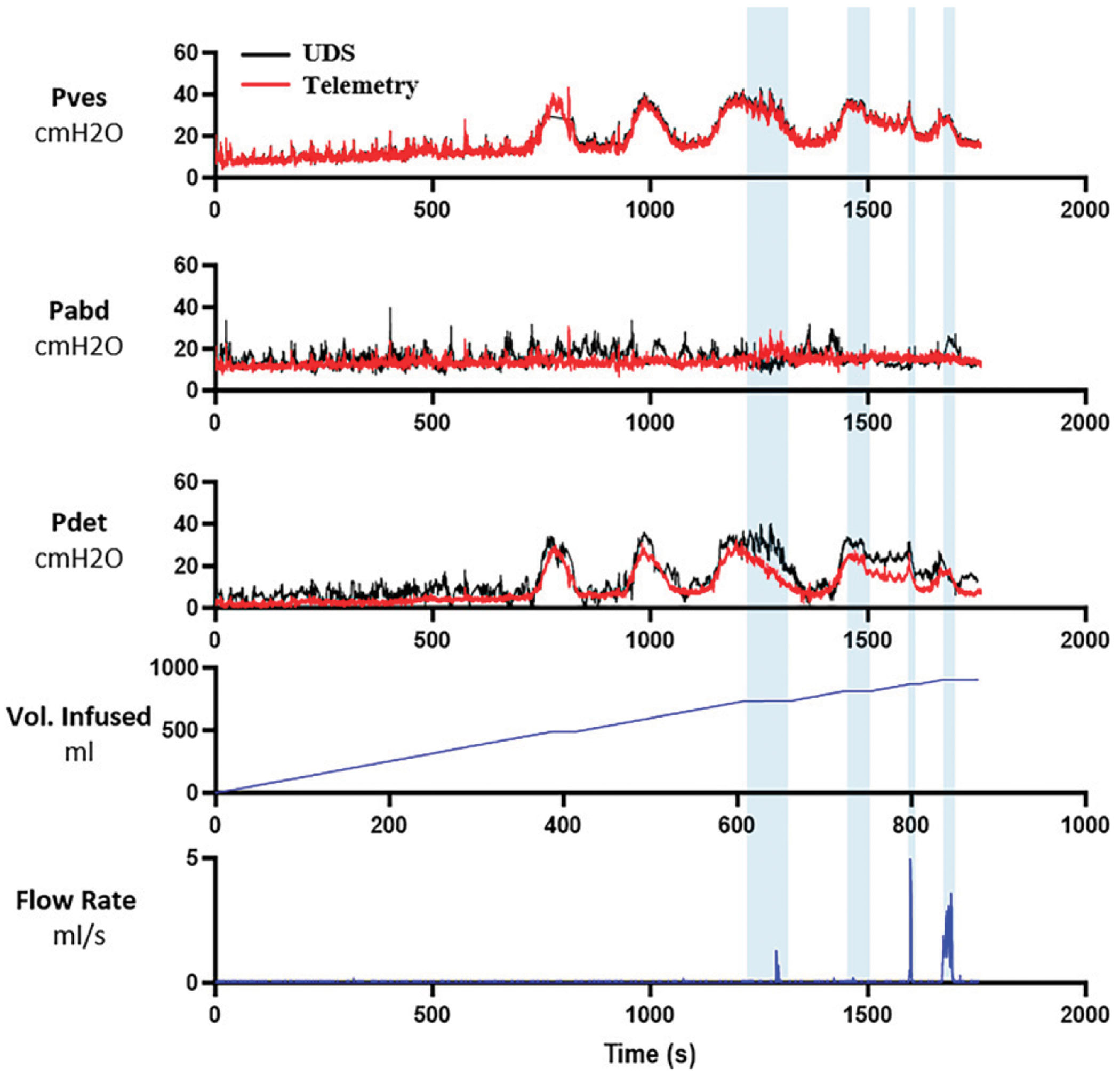
Example cystometrogram showing simultaneous air-charged catheter-based UDS bladder pressures (black) and telemetric transmural and intra-abdominal pressure sensors (red) in a spinal cord-injured Yucatan minipig 11 weeks after spinal cord injury (SCI). Four voiding contractions are seen herein and highlighted with blue shading. Simultaneous volume infused (mL) and voided urine flow rate (mL/sec) are also included (dark blue). Pves, bladder pressure; Pabd, abdominal pressure; Pdet, detrusor pressure (Pves – Pabd). UDS, urodynamic studies.

To evaluate the performance of the telemetry pressure sensors, we compared measured pressure data with filling and voiding pressures during conventional cystometry. Examples of simultaneous telemetry and catheter-based UDS pressure data recorded before (Fig. 4) and 11 weeks after SCI, 16–17 weeks after sensor implantation (Fig. 5), both show alignment of clinically relevant events. We quantitatively compared pressure data in 27 (75%) of the 36 cystometry experiments (9 animals × 4 cystometries each). We excluded bladder pressure data in nine sessions due to UDS catheter malfunctioning (*n* = 1; 13 weeks post-implantation), telemetry transmural sensor migrating from the bladder (*n* = 4), unresponsive telemetry transmural sensor (*n* = 2), unresponsive telemetry implant (*n* = 1; 17 weeks post-implantation), or extensive baseline drift (*n* = 1; 8 weeks post-implantation). Abdominal pressure data were excluded or unattainable in two sessions due to unresponsive telemetry implant (*n* = 1; 17 weeks post-implantation) or malfunctioning UDS catheter (n = 1; animal H; 14 weeks post-implantation).

**FIG. 6.**
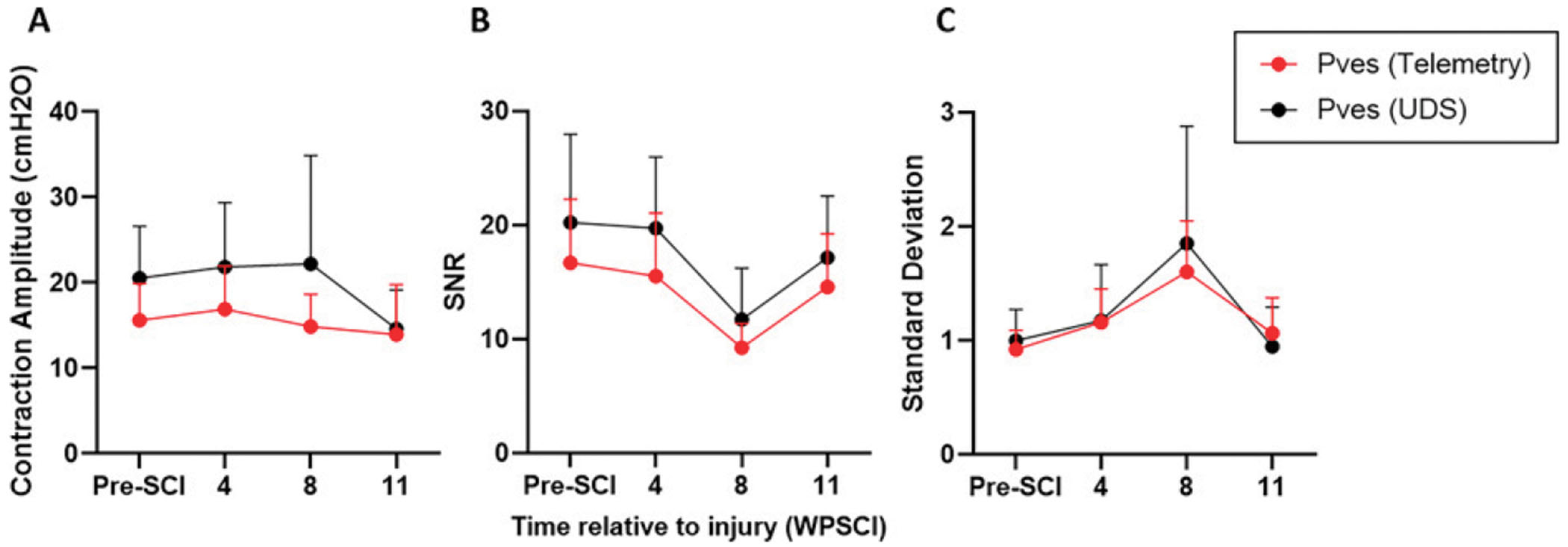
Quantitative outcomes comparing the performance of simultaneous bladder pressure measurements (Pves) using the telemetry system and the catheter-based urodynamic (UDS) system. *N* = 7 animals were included in this analysis. A total of 9 contractions were analyzed from uninjured (pre-SCI) animals, 30 contractions from 4 weeks post-SCI animals, 33 contractions at 8 weeks, and 13 contractions at 11 weeks post-SCI. *(A)* Voiding contraction amplitude; *(B)* signal-to-noise ratio (SNR); *(C)* standard deviation of pressure measurements within a 50 s window. (Telemetry = red; UDS = black). Data are presented as mean ± standard deviation. There were no significant differences between the data sets at each timepoint before or after spinal cord injury (SCI) as determined using a mixed-effects model analysis. WPSCI, weeks post spinal cord injury.

Voiding contraction amplitude demonstrated no significant difference between measurement modalities (*p* = 0.11) and no significant change with time after SCI (*p* = 0.09) (Fig. 6A). Signal-to-noise ratio (SNR) was assessed as a performance metric of bladder pressure measured with both the UDS catheter and the implanted transmural pressure sensor (Fig. 6B). There were no significant differences in SNR with pressure measurement modality (*p* = 0.36) or with time after SCI (*p* = 0.13), demonstrating similar fidelity and no degradation of signal performance. Similarly, the standard deviation of baseline pressure measurements showed no significant difference between measurement modalities (*p* = 0.55) (Fig. 6C), confirming similar fidelity and lack of degradation of signal performance.

**FIG. 7.**
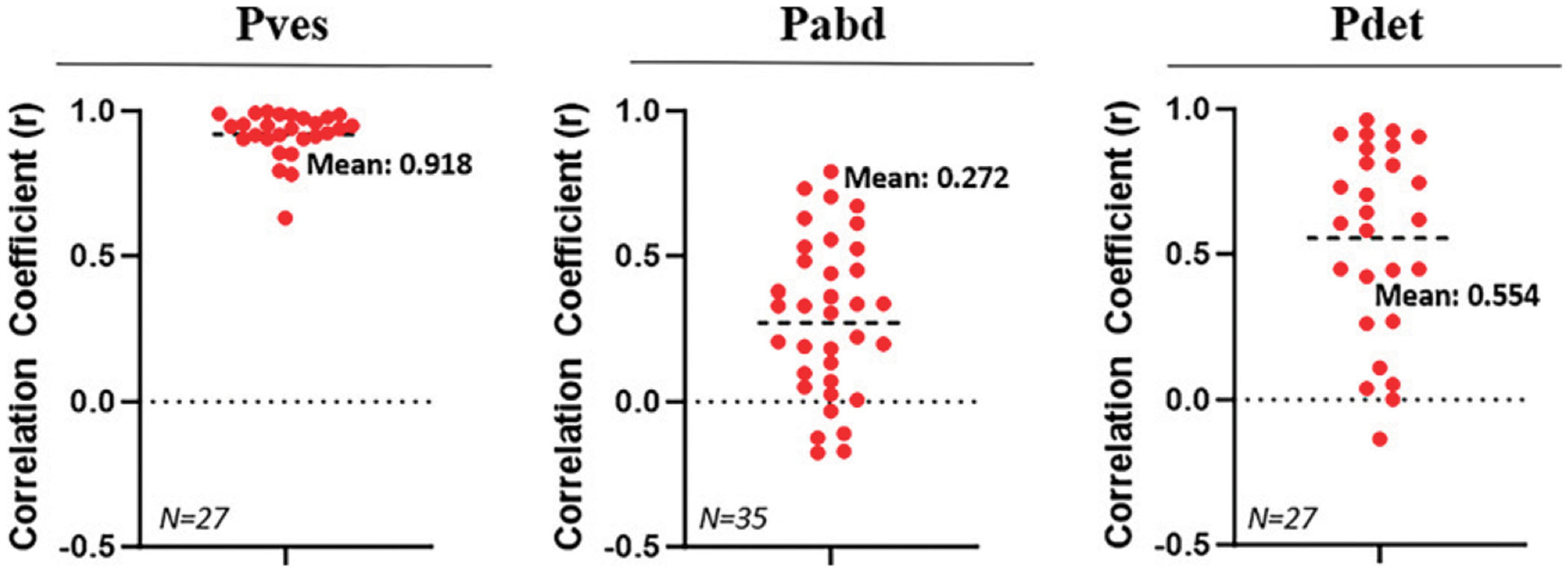
Correlation coefficients comparing simultaneously measured urodynamic air-charged catheters to the implanted telemetric pressure measurement system during cystometry. *N* = 8 animals were included in the present analysis. Pves, bladder pressure; Pabd, abdominal pressure; Pdet, detrusor pressure, which is calculated as Pves – Pabd.

Correlation coefficients between bladder, detrusor, and Pabds measured simultaneously with the two measurement modalities were calculated for each cystometry test (Fig. 7). Bladder pressure measurements correlated very closely between the two measurement modalities, with a mean ± standard deviation of the correlation coefficients of 0.92 ± 0.10. Measurements in subsequent weeks did not improve or worsen the bladder pressure correlation coefficients since bladder pressure correlation between sensors at the longest implantation period 17 weeks after SCI remained high (*r* = 0.92). Correlations between Pabd measured simultaneously by the two measurement modalities were weaker than for bladder pressure with a correlation coefficient of 0.27 ± 0.30. Rectal contractions were observed from the Pabd UDS catheter in 3 out of 35 (8.6%) experiments, which reduced the quality of the Pabd correlation. Pdet, calculated from bladder and Pabd, showed a moderate to strong correlation with a value of 0.55 ± 0.33.

**FIG. 8.**
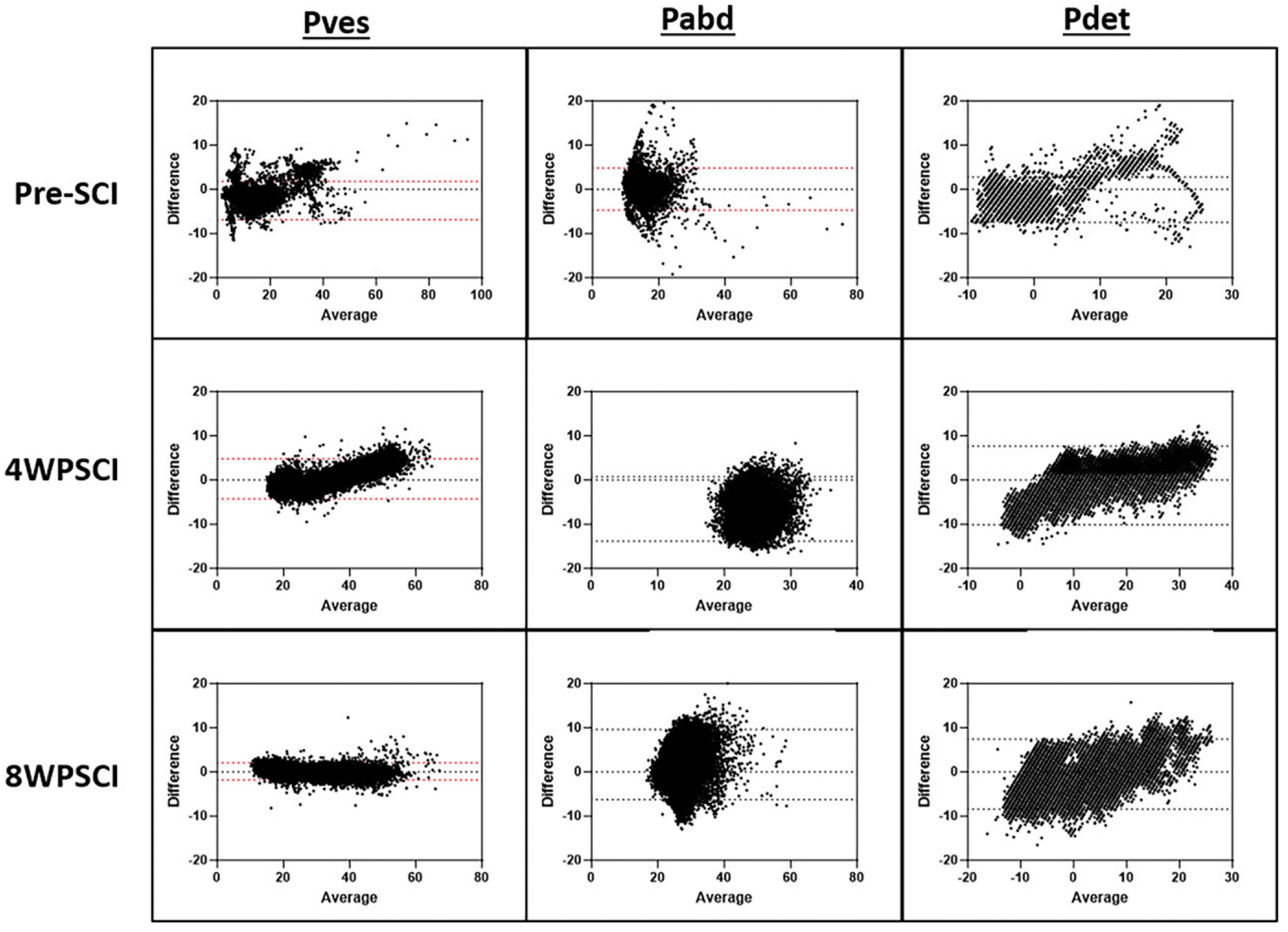
Bland–Altman analysis comparing Pves, Pabd, and Pdet pressure sensors from one spinal cord-injured (SCI) animal during cystometry at pre-SCI, 4 weeks post-injury, and 8 weeks post-injury. Difference (UDS − Telemetry) is presented on the y-axis, and average [(UDS + Telemetry)/2] is presented on the x-axis. Dotted lines represent the upper and lower limits of agreement (95% limits of agreement). Data sampling rate was adjusted such that UDS and telemetry values were exported at 20 Hz. Recording was performed from the beginning of bladder fill to the conclusion of the experiment. These findings demonstrate that the difference between intravesical pressure sensors (Pves UDS – Pves Telemetry) is generally within ±10 cmH_2_O, which is considered a strong agreement. The difference between abdominal pressure sensors (Pabd UDS – Pabd Telemetry) is generally within ±10 cmH_2_O but is sometimes outside of those boundaries, which is considered a mild agreement. The difference between detrusor pressure calculation (Pdet UDS – Pdet Telemetry) is generally within ±10 cmH_2_O but is sometimes outside of those boundaries, which is considered moderate to weak agreement based on parameters established in the present study.

Bland–Altman analysis was used to assess the degree of agreement between the UDS catheters and telemetry sensors (Fig. 8). The mean difference (bias) in bladder pressure measured simultaneously with the UDS and telemetry systems was 0.56 ± 5.1 cmH_2_O (95% confidence interval [CI]: −1.3 to 2.4), indicating that the absolute values measured with the UDS catheters were on average 0.56 cmH_2_O greater than those measured with the telemetry sensor, which is not a clinically significant difference. The bias in Pabd between the UDS and telemetry systems was −0.78 ± 6.2 cmH_2_O (95% CI: −2.95 to 1.07), indicating that the absolute values measured with the UDS system were on average 0.78 cmH_2_O lower than those measured with the telemetry system. The bias in Pdet between the UDS and telemetry systems was 0.9 ± 4.9 cmH_2_O, indicating the absolute values measured with UDS catheters were on average 0.9 cmH_2_O greater than those measured with telemetry. These results demonstrate that, on average, pressures measured with our telemetry system were within ±1 cmH_2_O of the UDS catheters for Pves, Pabd, and Pdet during cystometric assessment in minipigs.

### Contraction amplitudes and clinically relevant parameters as measured with UDS catheters and the telemetry sensor

**FIG. 9.**
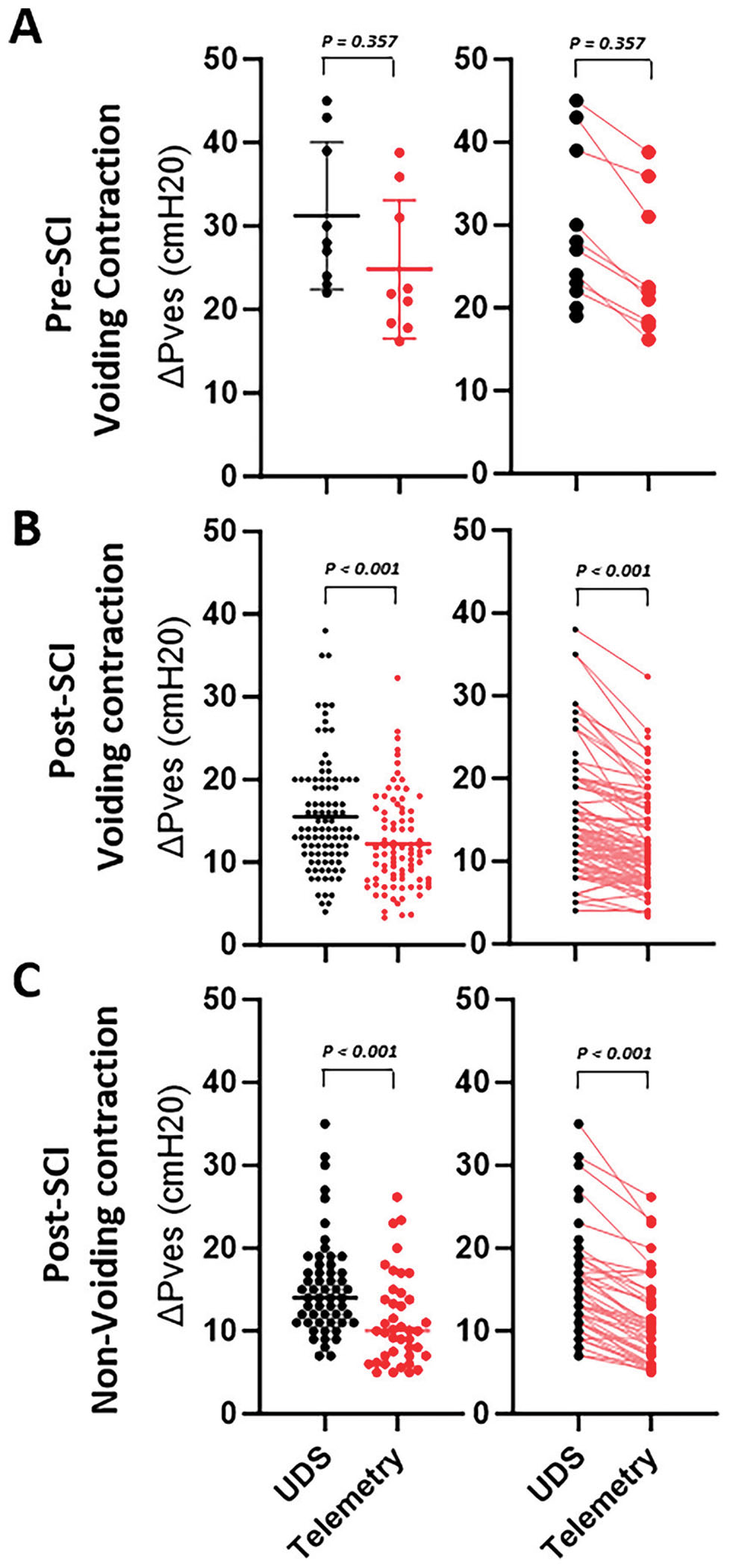
Voiding contraction analysis comparing conventional UDS air-charged catheter sensors (black) to telemetric transmural pressure recording (red) in Yucatan minipigs. *N* = 8 animals were included in the present analysis. **(A)** Pre-SCI voiding contraction amplitude; **(B)** Post-SCI voiding contraction amplitude; **(C)** Post-SCI non-voiding contraction amplitude. Each point represents the amplitude measurement of one voiding contraction as calculated from just prior to the contraction to the highest pressure value during the contraction. Telemetry = red; UDS = black. These results demonstrate that contraction amplitudes measured with the telemetry Pves sensor are generally 3–5 cmH20 lower than those measured with UDS Pves. These differences are considered statistically significant in post-SCI contractions. UDS, urodynamic studies.

Figure 9 demonstrates the contraction amplitude comparison between intravesical (Pves) pressure sensors. The mean voiding contraction pressure in the uninjured setting (Fig. 9A) was 31.2 ± 8.8 cmH_2_O from the UDS catheter and 24.8 ± 8.3 cmH_2_O from the telemetry sensor. Voiding contractions in the pre-SCI setting were on average 6.4 ± 2.8 cmH_2_O greater when measured with UDS catheters (paired two-tailed *t* test; *p* = 0.0001). The amplitude of post-SCI voiding contractions (Fig. 9B) was 15.3 ± 7.3 cmH_2_O from UDS catheters and 12.1 ± 5.6 cmH_2_O from the telemetry sensor. These differences were statistically significant (paired two-tailed *t* test; *p* < 0.0001). Mean of differences for post-SCI voiding contractions was 3.2 ± 3.2 cmH_2_O. Post-SCI non-voiding contractions (Fig. 9C) were 15.3 ± 7.4 cmH_2_O with UDS catheters and 11.3 ± 5.4 cmH_2_O with the telemetry sensor. These differences were statistically significant (paired two-tailed *t* test; *p* < 00001) with a mean difference of 4.1 ± 2.5 cmH_2_O.

**FIG. 10.**
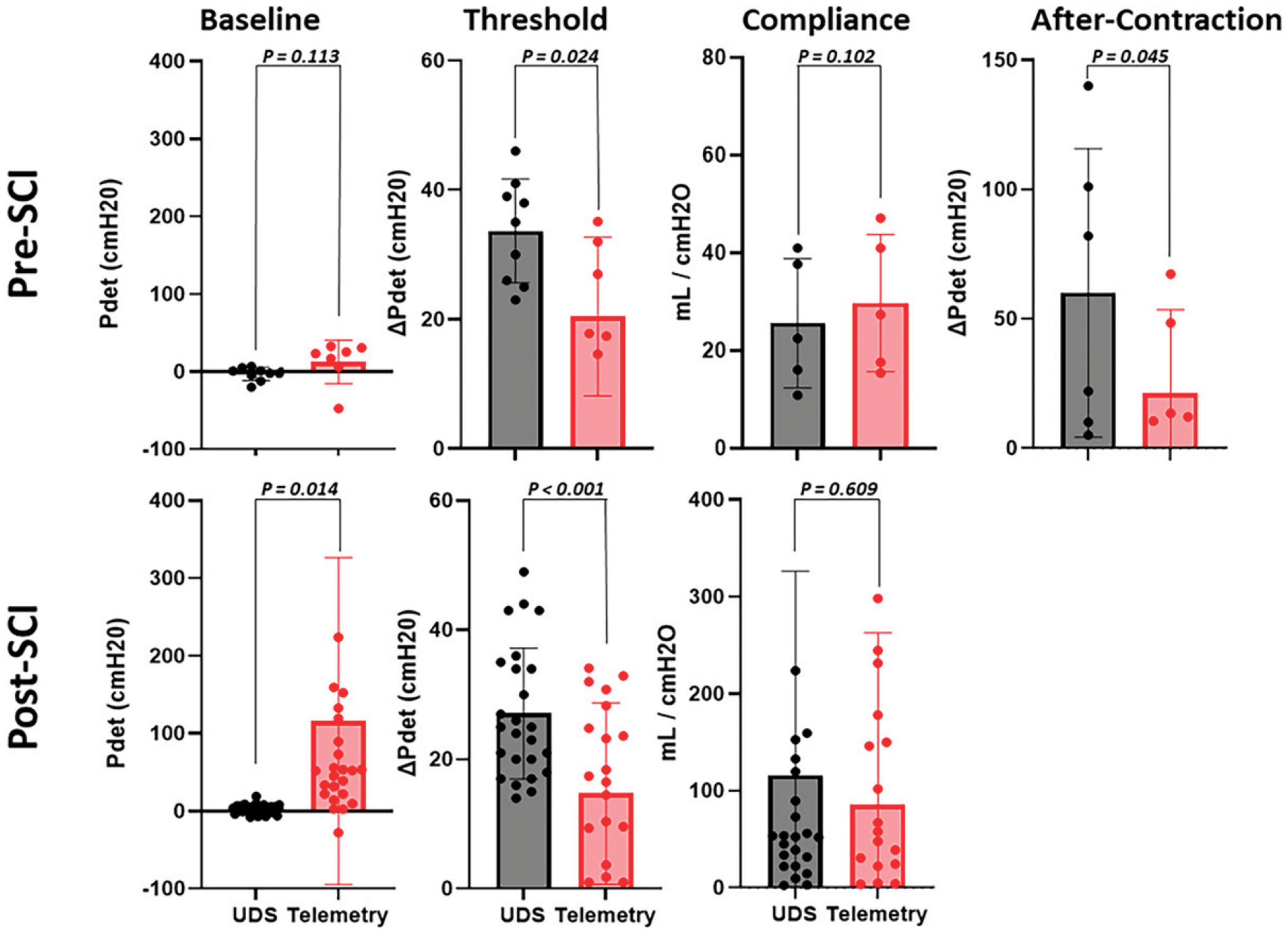
Comparison of clinically relevant parameters including baseline pressure, compliance, threshold pressure to void and detrusor after-contraction between pressure-sensing modalities in the pre and post-SCI setting. Differences were compared using a two-tailed paired t test. *N* = 8 animals were included in the present analysis. In uninjured pigs, one fill-and-void cycle was recorded. In SCI animals, which did not void volitionally, we continued the UDS recording until four leaks were observed. This explains the greater number of voids analyzed in post-SCI experiments. UDS = black, Telemetry = red. Detrusor after-contractions were not observed in post-SCI animals. UDS, urodynamic studies.

Having established the accuracy of the actual measurement of pressure by the telemetry system, we then assessed parameters that are typically measured in clinical UDS studies, including baseline pressure, compliance, threshold pressure to void, and the amplitude of detrusor after-contractions (Fig. 10).

Baseline Pdet pressure, measured at the initiation of bladder fill, was not statistically different between UDS catheters (1.6 ± 7.6 cmH_2_O) and the telemetry system (5.5 ± 47 cmH_2_O) (two-tailed; *p* = 0.654).

Compliance was calculated as the change in bladder volume (ΔV) divided by the change in bladder pressure (ΔPdet) as measured from the beginning of bladder fill to the point before the initiation of the first contraction waveform. Compliance values (*n* = 26) ranged significantly with a mean of 71.2 ± 98.2 mL/cmH_2_O as measured with UDS catheters and 56.2 ± 103.2 mL/cmH_2_O as measured using the telemetry sensors. Bladder compliance as measured with telemetry was not statistically significant from UDS catheters (*p* = 0.458).

Threshold pressure to void was calculated using the change in pressure (ΔPdet) from the beginning of bladder fill to the pressure at which a void started. Threshold pressure (*n* = 27) during cystometry experiments in pre- and post-SCI minipigs was 28.5 ± 10.1 cmH_2_O with UDS catheters and 15.6 ± 13.5 cmH_2_O with the telemetry sensors. The ~13 cmH_2_O (54.7%) average decrease in threshold pressure was statistically significant (paired two-tailed *t* test; *p* < 0.0001).

A “detrusor after-contraction” is defined in the ICS Glossary^25^ as a continued or new Pdet rise immediately after flow has ended. Herein, it was measured as the change in pressure (ΔPdet) before the initiation of the contraction to the maximum pressure noted during the after-contraction. After-contractions were seen exclusively in pre-SCI animals in the present study and were detected in six out of nine pre-SCI experiments. After-contraction amplitudes were greater when measured using UDS catheters (60 ± 55.7 cmH_2_O) relative to telemetry sensor measurements (23.6 ± 32 cmH_2_O). The mean difference in after-contraction amplitude between pressure sensors was not considered statistically significant in the present study (*p* = 0.068). A summary of all parameters typically used when assessing patients with NLUTD can be found in Table 2.

## Discussion

In this study, we implanted a novel telemetric bladder pressure monitoring system into a Yucatan pig model of SCI and compared its performance against clinical-grade UDS catheters. Both before and after thoracic SCI, the telemetry system accurately measured bladder pressure during filling cystometry and allowed for long-term, repeated monitoring of detrusor function.

Overall, the device was well-tolerated without any observable alterations to the voiding pattern (i.e., posture, flow, and/or void duration) or damage to bladder tissues, as confirmed with post-mortem histological analysis (not shown) including H&E and Masson’s trichrome staining. During the course of the study, spanning a period of 13–18 weeks post-implantation, all telemetry Pves sensors were observed to remain fully operational, with the exception of two instances. Furthermore, the Pabd sensor (*Pabd Caged Titanium*) exhibited responsiveness throughout the study and remained functional at the terminal end-point in all animals. This demonstrates that the telemetry system is highly durable over this time frame.

It is also worth noting that the standard deviation of baseline pressure data as calculated in the signal-to-noise analysis during this study did not demonstrate any significant variation between the two devices, indicating that there was little to no change in device performance relative to the clinical standard UDS catheters over the 13–18 weeks post-implantation period.

Despite its notable durability, the telemetry Pves pressure sensor in comparison to the UDS catheters consistently showed lower voiding and non-voiding contraction amplitudes, as well as a decrease in threshold pressure to void and detrusor after-contraction amplitude. These findings were consistent for both injured and non-injured scenarios. These results align with the study conducted by Wang and Chen in 2002, which demonstrated that, although not implanted through the detrusor wall, measurements procured from the micro-tip sensor were significantly lower when compared to those obtained using UDS fluid-filled catheters in women with genuine stress incontinence.^26^

Similarly, Zehnder et al.^27^ compared measurements of maximum urethral closure pressure using UDS air-charged catheters versus micro-tip pressure sensors in a prospective, single-blind, randomized trial. They concluded that micro-tip sensors were at least as reliable as the UDS catheters; however, the micro-tip sensors generally gave lower urethral pressure readings and could therefore not be used interchangeably for clinical purposes. Alternatively, it is important to recognize that the sensors were calibrated independently by different experimenters, potentially leading to varying sensitivities between the two devices.

Despite the lower voiding amplitudes, the study revealed a strong correlation between Pves pressure measurements obtained through the use of the telemetry sensor and the UDS catheter. This correlation is observed to be consistent across different animals and timepoints, thus indicating that the telemetric transmural pressure sensor can serve as a reliable and accurate alternative to the UDS catheter for monitoring bladder pressure.

Correlation coefficients comparing the telemetry abdominal sensor to the UDS catheter were weaker and more variable relative to Pves pressures. This could be due to the presence of characteristic pressure waveforms recorded from the UDS catheter as a result of rectal contractions in 8.5% of experiments. Rectal contractions are occasionally observed during human urodynamic assessment (~6.5% of examinations) and have been described in several studies.^28-31^ These contractions induce a significant flux in pressure within the rectal cavity and are detected by the UDS catheter. Given that the rectal pressure fluctuations are not present in the peritoneal cavity, where the telemetry abdominal sensors reside, the fluctuations in rectal pressure dramatically reduce the correlation and agreement between Pabd sensors.

Additionally, the difference in Pabd measurements may also be attributed to the fact that the sensors reside in different locations within the animal. Studies have indicated that rectal or vaginal pressure measurements can serve as a means to estimate intra-abdominal pressures during urodynamic evaluations^32-34^; however, one study demonstrated differences in pressure that could significantly alter the inference of Pdet.^35^ It should therefore be appreciated that the pressure values recorded outside of the abdominal cavity merely represent approximations of intra-abdominal pressure and are not definitive measures. Additionally, current evidence regarding the use of rectal pressure as a surrogate for intra-abdominal pressure is based on several studies in the fields of critical care, anesthesia, and surgery^36-39^ though not as extensively studied in the field of urology. Therefore, further investigation is warranted to establish a more comprehensive understanding of the relationship between rectal and intra-abdominal pressure during urodynamic assessment.

Simultaneous Pves and Pabd measurement from our wireless telemetry sensor enabled us to calculate Pdet pressure and compare it to the clinical standard assessment of Pdet during awake cystometry in minipigs. We observed variable correlation strengths from very strong to weak when comparing Pdet pressure as measured with the telemetry system and UDS catheters. We suspect that the correlation between these two techniques was affected primarily by the performance of the telemetry Pabd sensor, as mentioned above. When assessing the agreement between Pdet pressure values from the two systems, the Bland–Altman analysis showed an overall strong agreement between sensors. Clinically relevant measurements using Pdet assessed herein included baseline pressure, compliance, threshold pressure to void, and after-contraction amplitude. We found there was no significant difference between baseline pressure measurements and compliance in the present study. However, we did observe significantly lower values of threshold pressure to void and detrusor after-contraction amplitude as measured with the wireless telemetry sensors. We believe that the decreased sensitivity of the transmural telemetry sensor (as noted with the significantly lower amplitude of voiding and non-voiding contractions) contributed to the lower threshold pressures to void and detrusor after-contraction amplitudes. These findings demonstrate that the values obtained with our telemetry pressure (Pves) sensors were approximately within 3–4 cmH_2_O; however, those obtained using Pdet measurement showed more variability relative to UDS catheters.

In the present study, we included an assessment of the detrusor after-contraction (the continued or new Pdet rise after the flow has ceased during micturition) as a clinically relevant parameter. However, detrusor after-contraction is not presently considered a relevant parameter when assessing subjects with NLUTD,^40^ and it is still unclear whether it is truly a physiological phenomenon or merely a recording artefact.^41,42^ Valentini et al. concluded that the most reasonable explanation underlying the after-contraction is a recording artifact as a result of the pressure sensor contacting the bladder wall toward the end of a void at low volume. In the present study, we observed this phenomenon in both the UDS catheters and the telemetry transmural pressure probe in six out of nine pre-SCI animals. Detrusor after-contraction was not observed in any post-SCI animals as measured by the two pressure sensors. After-contractions measured with the UDS catheter were significantly greater than those measured with the telemetry transmural sensor. In addition, the mean difference was approximately 30 cmH_2_O, which is much greater than the average difference between voiding contraction amplitudes (~3–4 cmH_2_O). Consistent with the interpretation of Valentini et al., we also believe that the detrusor after-contraction is a recording artifact resulting from the sensor making contact with the bladder wall. This would explain why the after-contraction was much greater with the UDS catheter (which can be pushed up against the bladder wall) than with the telemetry system, where the Pves sensor that protrudes into the lumen (away from the wall) by ~1.5 cm.

In a related study, Huppertz et al. 2015^22^ investigated the voiding behavior of Gottingen minipigs using a telemetric transmural Pves pressure sensor. Interestingly, they recorded mean voiding contraction amplitudes (ΔPdet) of ~100 cmH_2_O in free-moving and restrained animals. However, it is conceivable that the maximum amplitudes measured in their study were actually detrusor after-contractions that were apparent at the termination of the void. We hypothesize that the larger after-contraction amplitudes recorded in their study could be explained by differences in transmural pressure probe length (though no pressure sensor measurement was presented in the article) and/or differences related to calibration.

## Conclusion

The findings of this study demonstrate the potential of this radiotelemetry system to perform reliable and long-term monitoring of bladder pressure in SCI pigs. When compared to air-charged catheters, the transmural pressure sensor showed a strong correlation of pressure data and was able to measure the amplitude of bladder contractions within 5 cmH_2_O. However, significant differences were noted when comparing the measurement of UDS parameters relative to conventional catheters, which are likely related to the incongruence of abdominal pressure sensing from different locations in the animal. If such telemetric techniques can be applied to other animal models of SCI, this would represent a unique tool for *in vivo* pharmacology studies and for a better understanding of LUT function and its dysfunction after neurological injury.

### Transparency, Rigor, and Reproducibility Statement

This study was conducted with a commitment to transparency, scientific rigor, and reproducibility to ensure the reliability of its findings and their utility for the broader research community.

We provide detailed descriptions of all experimental protocols, including surgical procedures for telemetry device implantation, animal care and training regimens, cystometric assessments, and SCI induction. All telemetry system specifications and calibration procedures are clearly described to allow replication. Data processing techniques, such as down-sampling, baseline drift correction, and criteria for contraction identification, are explicitly outlined. Key limitations, including pressure sensor migration, signal drift, and differences in pressure measurement locations, are acknowledged and discussed.

Rigorous scientific practices were followed throughout the study. All experimental procedures were approved by the Animal Care Committee at UBC and conformed to CCAC guidelines. Controls were included through simultaneous conventional UDS recordings to directly compare performance with the telemetry system. Statistical methods were chosen based on appropriate assumptions and included Pearson correlation, Bland–Altman analysis, and mixed-effects modeling to evaluate agreement, variance, and signal fidelity. We also identified and excluded data where pressure sensors failed or calibration drifted beyond acceptable limits, minimizing bias.

To promote reproducibility, we used commercially available telemetry systems (TSE Systems) with standardized calibration. All urodynamic testing was performed using a consistent protocol, including sling training and standardized sedation and infusion rates. The criteria for data inclusion/exclusion and analytical thresholds (e.g., contraction amplitudes >7 cm H_2_O) are clearly stated. Statistical analyses were performed using widely used software (GraphPad Prism and MATLAB), and parameters like signal-to-noise ratio were computed using defined algorithms.

By embedding these principles into our study design and reporting, we aim to provide a reliable foundation for future research into neurogenic bladder dysfunction and to support the broader adoption of wireless bladder pressure monitoring in pre-clinical models of SCI.

## Figures and Tables

**Table 1.** Experimental Spinal Cord Injury Parameters

Animal name	Weight (kg)	Impulse (kdyne*sec)	Vcontact (mm/sec)	Displacement (mm)	Δh/Vcontact (sec)	Zeroed force (kdyne)
A	28.2	10.0	1683.9	4.3	0.0026	3070.5
B	26.6	10.2	1669.6	4.5	0.0027	2625.0
C	26.6	9.6	1680.5	5.1	0.0030	2410.4
D	30.1	9.8	1678.7	5.0	0.0030	2640.7
E	27.6	10.1	1677.1	4.6	0.0027	2889.5
F	28.2	10.2	1689.3	4.7	0.0028	2768.4
G	25.8	10.5	1682.2	4.7	0.0028	2782.6
H	27.6	10.1	1704.0	5.6	0.0033	2382.1
I	28.0	10.0	1667.8	4.7	0.0028	2785.0

All animals received a contusion/compression spinal cord injury (SCI) using a 50 g impactor at the T10 level from 16.73 cm. Animal weight is listed along with various injury parameters including impulse (kdyne*sec), velocity at contact [Vcontact (mm/sec)], displacement (mm), ratio of displacement relative to velocity at contact [Δh/Vcontact (sec)], and zeroed force (kdyne).

**Table 2.** Summary of Data Inclusion and Exclusion Across Experimental Timepoints for Individual Animals

		Post-SCI data			
Animal ID	Pre-SCI data	4 weeks	8 weeks	11 weeks	Reason for exclusion (if applicable)
A	✓	✓	✓	✗	Telemetry bladder sensor malfunction at 11 weeks post-SCI
B	✓	✓	✓*	✓**	*UDS performed at 7 weeks post-SCI due to UTI complications
					**UDS performed at 7.5 weeks post-SCI due to UTI complications
C	✗	✓	✓*	✓**	Telemetry bladder sensor failure pre-SCI; replacement sensor used post-SCI *UDS performed at 7 weeks post-SCI due to UTI complications **UDS performed at 7.5 weeks post-SCI due to UTI complications
D	✗	✗	✗	✗	Telemetry bladder sensor migration out of the bladder lumen Abdominal pressure (Pabd) data are still used in several analyses
E	✓	✗	✓	✓	Supraphysiological pressure drift at 4 weeks post-SCI
F	✓	✓	✓	✓	
G	✓	✓	✓	✗***	Telemetry bladder sensor malfunction at 8.5 weeks post-SCI
					***UDS performed at 8.5 weeks post-SCI due to UTI complications
H	✓	✓	✓	✓	
I	✓	✓	✓	✓	

Pre-SCI and post-SCI urodynamic (UDS) data availability is shown for each animal pre-SCI and at 4, 8, and 11 weeks post-SCI. Check marks indicate data included in analyses, while crosses indicate exclusion. Reasons for data exclusion are provided where applicable, including telemetry bladder sensor failure, migration, or malfunction. Abdominal pressure (Pabd) data from excluded animals were retained for analyses where feasible.
